# Effect of chlorhexidine Mouthrinse on prevention of microbial contamination during EBUS-TBNA: a randomized controlled trial

**DOI:** 10.1186/s12885-022-10442-5

**Published:** 2022-12-20

**Authors:** Na Young Kim, Jae Hyeon Park, Jimyung Park, Nakwon Kwak, Sun Mi Choi, Young Sik Park, Chang-Hoon Lee, Jaeyoung Cho

**Affiliations:** 1 Division of Pulmonary and Critical Care Medicine, Department of Internal Medicine, Seoul National University Hospital, Seoul, Republic of Korea; 2 Department of Laboratory Medicine, Seoul National University Hospital, Seoul, Republic of Korea; 3 Department of Laboratory Medicine, Seoul National University College of Medicine, Seoul, Republic of Korea; 4 Department of Internal Medicine, Seoul National University College of Medicine, 101 Daehak-ro, Jongno-gu, Seoul, 03080 Republic of Korea

**Keywords:** Chlorhexidine gluconate, EBUS-TBNA, Infectious complication, Mouthrinse

## Abstract

**Background:**

Although endobronchial ultrasound-guided transbronchial needle aspiration (EBUS-TBNA) is a minimally invasive procedure, fatal infectious complications have been reported. However, adequate preventive strategies have not been determined. We aimed to investigate the effect of chlorhexidine mouthrinse on the prevention of microbial contamination during EBUS-TBNA.

**Methods:**

In this single-center, assessor-blinded, parallel-group randomized controlled trial, we randomly assigned adult participants undergoing EBUS-TBNA using a convex probe to gargle for 1 minute with 100 mL of 0.12% chlorhexidine gluconate before EBUS-TBNA or to receive usual care (no chlorhexidine mouthrinse). Aspiration needle wash samples were collected immediately after completion of EBUS-TBNA by instilling sterile saline into the used needle. The primary outcome was colony forming unit (CFU) counts per mL of needle wash samples in aerobic cultures. Secondary outcomes were CFU counts per mL of needle wash samples in anaerobic cultures, fever within 24 hours after EBUS-TBNA, and infectious complications within 4 weeks after EBUS-TBNA.

**Results:**

From January 2021 to June 2021, 106 patients received either chlorhexidine mouthrinse (*n* = 51) or usual care (*n* = 55). The median CFU counts of needle wash samples in aerobic cultures were not significantly different in the two groups (10 CFU/mL vs 20 CFU/mL; *P* = 0.70). There were no significant differences between the groups regarding secondary outcomes, including median CFU counts in anaerobic cultures (*P* = 0.41) and fever within 24 hours after EBUS-TBNA (11.8% vs 5.6%, *P* = 0.31). There were no infectious complications within 4 weeks in both groups.

**Conclusions:**

Chlorhexidine mouthrinse did not reduce CFU counts in needle wash samples of EBUS-TBNA.

**Trial registration:**

ClinicalTrials.gov, NCT04718922. Registered on 22/01/2021.

**Supplementary Information:**

The online version contains supplementary material available at 10.1186/s12885-022-10442-5.

## Introduction

Endobronchial ultrasound-guided transbronchial needle aspiration (EBUS-TBNA) is the standard procedure for the diagnosis of mediastinal and hilar lymphadenopathy as well as the staging of lung cancer [1]. EBUS-TBNA is a minimally invasive and safe procedure, although infectious complications have been reported with its widespread use [2–8]. The incidence of infectious complications following EBUS-TBNA ranges from 0.19 to 0.48% [9, 10]. Although infrequent, infectious complications including mediastinitis, pericarditis, and sepsis can be fatal. Our institution previously reported four cases of infectious complications after EBUS-TBNA (two cases of mediastinal adenitis [11] and two cases of bacterial pericarditis [12]), one of which resulted in death.

Despite concerns about serious infectious complications associated with EBUS-TBNA, there are no established strategies to prevent such complications following this procedure. We hypothesized that oral hygiene care is important in preventing infectious complications during the procedure because previous studies have suggested that oropharyngeal commensal bacteria can contaminate the working channel of an EBUS bronchoscope and thus can be inoculated into the target lesion by a contaminated aspiration needle [13, 14]. Moreover, a recent retrospective study suggested that endobronchial intubation may prevent contamination by oropharyngeal commensal bacteria during EBUS-TBNA [15]. We conducted a randomized controlled trial (RCT) to evaluate whether mouthrinse with chlorhexidine, a broad-spectrum antimicrobial agent, reduces microbial contamination during EBUS-TBNA.

## Methods

### Study design and participants

This single-center, assessor-blinded, parallel-group RCT was performed from January 2021 to June 2021 at Seoul National University Hospital in South Korea. Adults (≥19 years old) who were hospitalized for EBUS-TBNA using a convex probe were randomly assigned to either the chlorhexidine mouthrinse group or the control group at a 1:1 ratio. Simple randomization was performed by a web-based randomization system developed and administered by the Medical Research Collaborating Center of the Seoul National University Hospital. A more detailed study protocol was published elsewhere [11]. Key exclusion criteria were: antiseptic mouthrinse within a week before EBUS-TBNA; overt infection or use of antibiotics within a week before EBUS-TBNA; immunocompromised status; and tracheostomy status.

### Procedures

A detailed description of the procedures was published previously [11]. In brief, all patients received topical oropharyngeal anesthesia with 20 mL of 1% lidocaine. After anesthesia, patients assigned to the intervention group gargled 100 mL of 0.12% chlorhexidine gluconate for 1 minute under the supervision and the direction of a nurse. Patients assigned to the control group were not provided with mouthrinse. Under conscious sedation, endobronchial evaluation was routinely performed using conventional flexible bronchoscopy unless it had already been conducted within the preceding several days. During conventional bronchoscopy, bronchial washing, bronchoalveolar lavage, endobronchial biopsy, and transbronchial lung biopsy were performed as required.

An EBUS bronchoscope with a convex probe (BF-UC260FW; Olympus, Tokyo, Japan) was used for EBUS-TBNA and was inserted orally. Following mediastinal evaluation using EBUS-TBNA was performed at the designated lymph nodes (LNs) or masses with a dedicated 22-gauge aspiration needle (NA-201SX-4022 or NA-U401SX-402; Olympus). At the bronchoscopist’s discretion, replacement of the aspiration needle with a new one, transesophageal bronchoscopic ultrasound-guided fine-needle aspiration (EUS-B-FNA), or rapid on-site evaluation of aspirates could be performed. Prescription of prophylactic antibiotics after EBUS-TBNA was also allowed as determined by the bronchoscopist or care provider.

As soon as EBUS-TBNA was completed, a needle wash sample was obtained by injecting 5 mL of sterile saline into the used needle. This sampling method has been described previously [8, 15]. If two or more needles were used during the procedure, each needle wash sample was collected separately. The needle wash samples were dispensed on aerobic and anaerobic media and cultured according to a routine clinical protocol. The bacteria were identified by matrix-assisted laser desorption ionization time-of-flight mass spectrometry (MALDI-TOF MS) using MALDI Biotyper (Bruker Daltonics, Bremen, Germany) with 6903 main spectra library. MALDI-TOF MS identifications were classified using score values proposed by the manufacturer: a score value of ≥2 indicated species identification, a score value between 1.7 and 1.999 indicated genus identification, and a score value of < 1.7 indicated unreliable identification.

### Outcome assessment

The primary outcome was colony forming unit (CFU) counts per milliliter of aspiration needle wash samples in aerobic culture. The secondary outcomes were CFU counts per milliliter of the needle wash samples in anaerobic culture, fever within 24 hours following EBUS-TBNA, and infectious complications within 4 weeks after EBUS-TBNA. If two or more needle wash samples were present in a patient, the average of CFU counts on each agar plate was considered as an outcome measure. Fever was defined as a body temperature of 37.8 °C or higher. Infectious complications were defined as mediastinal adenitis, mediastinal abscess, mediastinitis, pneumonia, lung abscess, empyema, pericarditis, and sepsis.

### Sample size calculation

The sample size was estimated using the results obtained from an RCT of 100 patients who underwent gastroscopy with or without chlorhexidine mouthrinse [16] because previous studies on CFU counts of TBNA needle wash samples were not available. A sample size of 50 participants per group was required to evaluate whether chlorhexidine mouthrinse reduces the CFU counts of needle wash samples by 50% with an alpha of 0.05 and a power of 90%.

### Statistical analysis

Data were analyzed according to the randomized allocation, excluding patients who withdrew consent or did not undergo EBUS-TBNA. Categorical variables are presented as counts and percentages. Continuous variables are presented as means with SD or median with interquartile range. CFU counts were analyzed by Mann–Whitney *U* test. We also estimated 95% CIs for median differences of CFU counts between the groups based on 1000 bootstrap replications and the percent method [17]. Secondary outcomes except CFU counts were analyzed by Fisher’s exact test. A sensitivity analysis was conducted in patients who underwent the whole procedure with one aspiration needle. Subgroup analyses were conducted according to age (≥70 years vs < 70 years), sex, smoking status (never vs ever smoker), and the presence of diabetes. All comparisons were two-sided, and *P* values of less than 0.05 were considered statistically significant. All analyses were performed with R version 4.1.1.

## Results

### Characteristics of participants and procedure

**Fig. 1:**
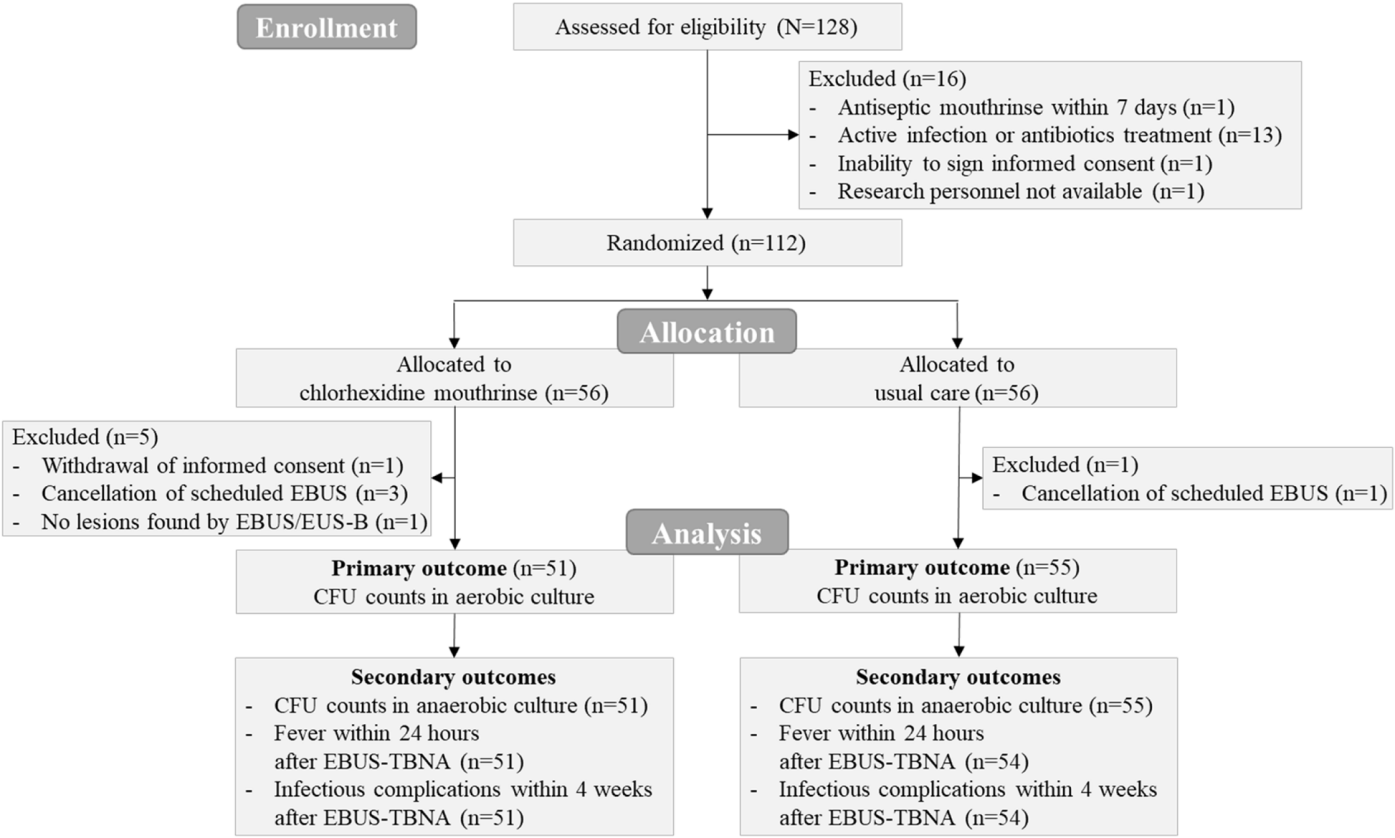
Consolidated Standards of Reporting Trials flowchart. CFU = colony forming unit; EBUS = endobronchial ultrasound; EBUS-TBNA = EBUS-guided transbronchial needle aspiration; EUS-B = transesophageal bronchoscopic ultrasound

Of 128 patients assessed for eligibility, 112 were randomly assigned to either the chlorhexidine mouthrinse group or the usual care group. After randomization and allocation, six patients were excluded from the study for reasons of withdrawal of informed consent in one, cancellation of scheduled EBUS following successful endobronchial biopsy in four, and no targeting lesions found by EBUS or EUS-B in one (Fig. 1). Thus, 106 patients (51 in the chlorhexidine mouthrinse group and 55 in the usual care group) were included in the primary analysis. The median age of the patients was 70 years, 76.4% of whom were men. Baseline characteristics were balanced between the two groups (Table 1). About half of the patients had hypertension and a quarter had diabetes. The most frequent final diagnosis was primary lung cancer (80.2%).

**Table 1:** Clinical Characteristics of the Study Participants

	Chlorhexidine Mouthrinse (*n* = 51)	Usual Care (*n* = 55)
Age, years	69 (63–78)	71 (65–78)
Male	40 (78.4)	41 (74.5)
BMI, kg/m^2^	23.0 ± 3.1	23.6 ± 2.8
Smoking status		
Never	13 (25.5)	19 (34.5)
Former	20 (39.2)	23 (41.8)
Current	18 (35.3)	13 (23.6)
Smoking intensity, pack-years	22.5 (0.5–40.0)	29.0 (0.0–40.0)
Comorbidities		
Hypertension	24 (47.1)	24 (43.6)
Diabetes	11 (21.6)	15 (27.3)
Coronary heart disease	3 (5.9)	9 (16.4)
Congestive heart failure	0 (0.0)	1 (1.8)
Stroke	4 (7.8)	1 (1.8)
Chronic kidney disease	4 (7.8)	3 (5.5)
Chronic liver disease	1 (2.0)	2 (3.6)
COPD	5 (9.8)	3 (5.5)
Asthma	1 (2.0)	0 (0.0)
Final diagnosis^a^		
Malignant	45 (88.2)	47 (85.5)
Primary lung cancer	42 (82.4)	43 (78.2)
Adenocarcinoma	20 (39.2)	23 (41.8)
Squamous cell carcinoma	15 (29.4)	15 (27.3)
Others^b^	2 (3.9)	3 (5.5)
Small cell carcinoma	5 (9.8)	2 (3.6)
Metastatic tumors	3 (5.9)	3 (5.5)
Malignant mesothelioma	0 (0.0)	1 (1.8)
Benign	6 (11.8)	6 (10.9)
Chronic granulomatous inflammation^c^	3 (5.9)	2 (3.6)
Castleman disease	1 (2.0)	1 (1.8)
Normal lymphoid tissue	2 (3.9)	3 (5.5)
Undiagnosed	0 (0.0)	2 (3.6)

Data are presented as number (%), mean ± SD, or median (interquartile range). *BMI* Body mass index, *COPD* Chronic obstructive pulmonary disease

^a^The final diagnosis of a participant was based on the pathologic results of endobronchial ultrasound-guided transbronchial needle aspiration (EBUS-TBNA) or transesophageal bronchoscopic ultrasound-guided fine-needle aspiration (EUS-B-FNA), percutaneous biopsies of primary or metastatic lesions, or surgical resection with mediastinal lymph node dissection. ^b^Includes two participants with adenosquamous carcinoma, one with non-small cell carcinoma not otherwise specified, one with large cell carcinoma, and one with mucoepidermoid carcinoma. ^c^Includes three participants with sarcoidosis and one with pulmonary tuberculosis

Table 2 shows procedural characteristics on a per-patient basis. These characteristics were not significantly different between the two groups except that the proportion of the patients with LNs with coagulation necrosis sign on ultrasound or aspirates with pus-like material was significantly higher in the chlorhexidine mouthrinse group (19.6% vs 5.5%; *P* = 0.03). The proportion of patients with LNs with heterogeneous echogenicity on ultrasound was not significantly different between the two groups (43.1% vs 29.1%; *P* = 0.13). More than 80% of the patients underwent conventional flexible bronchoscopy before EBUS-TBNA and less than 10% underwent EUS-B-FNA. In the majority of patients, the number of insertions of EBUS bronchoscope was one and the number of aspiration needles used per patient was one. About one-third of patients were prescribed prophylactic antibiotics after EBUS-TBNA.

**Table 2:** Procedural Characteristics (Per-Patient Analysis)

	Chlorhexidine Mouthrinse (*n* = 51)	Usual Care (*n* = 55)	*P* Value
Indication for EBUS-TBNA			> 0.99
Diagnosis or staging of malignancy	49 (96.1)	52 (94.5)	
Diagnosis of benign disease	2 (3.9)	3 (5.5)	
Conventional bronchoscopy	42 (82.4)	49 (89.1)	0.32
Bronchial washing	6 (11.8)	5 (9.1)	0.65
Bronchoalveolar lavage	0 (0.0)	1 (1.8)	> 0.99
Endobronchial biopsy	8 (15.7)	3 (5.5)	0.08
EUS-B-FNA	4 (7.8)	5 (9.1)	> 0.99
Number of insertions of EBUS bronchoscope			0.50
≤ 1	45 (88.2)	46 (83.6)	
> 1	6 (11.8)	9 (16.4)	
Number of aspiration needles used per patient			0.28
≤ 1	44 (86.3)	51 (92.7)	
> 1	7 (13.7)	4 (7.3)	
Number of LNs punctured per patient			0.91
≤ 3	31 (60.8)	34 (61.8)	
> 3	20 (39.2)	21 (38.2)	
Total number of aspirations per patient			0.89
≤ 5	29 (56.9)	32 (58.2)	
> 5	22 (43.1)	23 (41.8)	
Characteristics of LNs/masses			
Heterogeneous echogenicity on ultrasound	22 (43.1)	16 (29.1)	0.13
Coagulation necrosis sign on ultrasound or aspirates with pus-like material	10 (19.6)	3 (5.5)	0.03
Procedure time			
Conventional bronchoscopy, min	3 (2–6)	3 (2–5)	0.56
EBUS-TBNA, min	19 (9–30)	20 (14–31)	0.37
Dosage of sedatives			
Midazolam, mg	4 (3–5)	5 (3–5)	0.37
Fentanyl, μg	50 (50–50)	50 (50–50)	0.49
Antibiotic prophylaxis after EBUS-TBNA	19 (37.3)	19 (34.5)	0.77

Data are presented as number (%) or median (interquartile range). *EBUS* Endobronchial ultrasound, *EBUS-TBNA* EBUS-guided transbronchial needle aspiration, *EUS-B-FNA* Transesophageal bronchoscopic ultrasound-guided fine-needle aspiration, *LN* Lymph node

The characteristics of 328 LNs or masses sampled in the study are summarized in Table 3. LNs or masses larger than 1 cm were more frequently found in the chlorhexidine mouthrinse group than in the usual care group (35.2% vs 22.5%; *P* = 0.01). The proportion of those with heterogeneous echogenicity on ultrasound was significantly higher in the chlorhexidine mouthrinse group (22.6% vs 11.8%; *P* = 0.01). Cytopathology examinations revealed that 24.5 and 17.9% of the LNs or masses were malignant in the chlorhexidine group and usual care group, respectively. The diagnostic accuracy of EBUS-TBNA in detecting nodal metastasis was 91% (95% CI, 82–96%; e-Table 1).

**Table 3:** Characteristics of Lymph Nodes and Masses (*N* = 328)

	Chlorhexidine Mouthrinse (*n* = 159)	Usual Care (*n* = 169)	*P* Value
LN stations/masses			
2R	14 (8.8)	17 (10.1)	
2L	0 (0.0)	1 (0.6)	
3P	0 (0.0)	1 (0.6)	
4R	40 (25.2)	42 (24.9)	
4L	29 (18.2)	29 (17.2)	
7	39 (24.5)	43 (25.4)	
10R	2 (1.3)	1 (0.6)	
10L	4 (2.5)	1 (0.6)	
11R	17 (10.7)	22 (13.0)	
11L	10 (6.3)	11 (6.5)	
Mass	4 (2.5)	1 (0.6)	
Number of aspirations per LN/mass			0.30
≤ 1	89 (56.0)	85 (50.3)	
> 1	70 (44.0)	84 (49.7)	
Ultrasound characteristics			
Size > 1 cm	56 (35.2)	38 (22.5)	0.01
Round shape	21 (13.2)	20 (11.8)	0.71
Distinct margin	123 (77.4)	122 (72.2)	0.28
Central hilar structure	29 (18.2)	46 (27.2)	0.05
Calcification	16 (10.1)	29 (17.2)	0.06
Heterogeneous echogenicity	36 (22.6)	20 (11.8)	0.01
Coagulation necrosis sign	10 (6.3)	6 (3.6)	0.25
Cystic lesion	0 (0.0)	0 (0.0)	
Gross visual appearance of aspirates			
Pus-like material	8 (5.0)	2 (1.2)	0.06
Cytopathology of LN/mass (*n* = 327)^a^			0.14
Malignant	39 (24.5)	30 (17.9)	
Benign	120 (75.5)	138 (82.1)	

Data are presented as number (%). *LN* Lymph node

^a^Endobronchial ultrasound-guided transbronchial needle aspiration for one LN failed to acquire cytopathologic specimens

### Outcomes

**Fig. 2:**
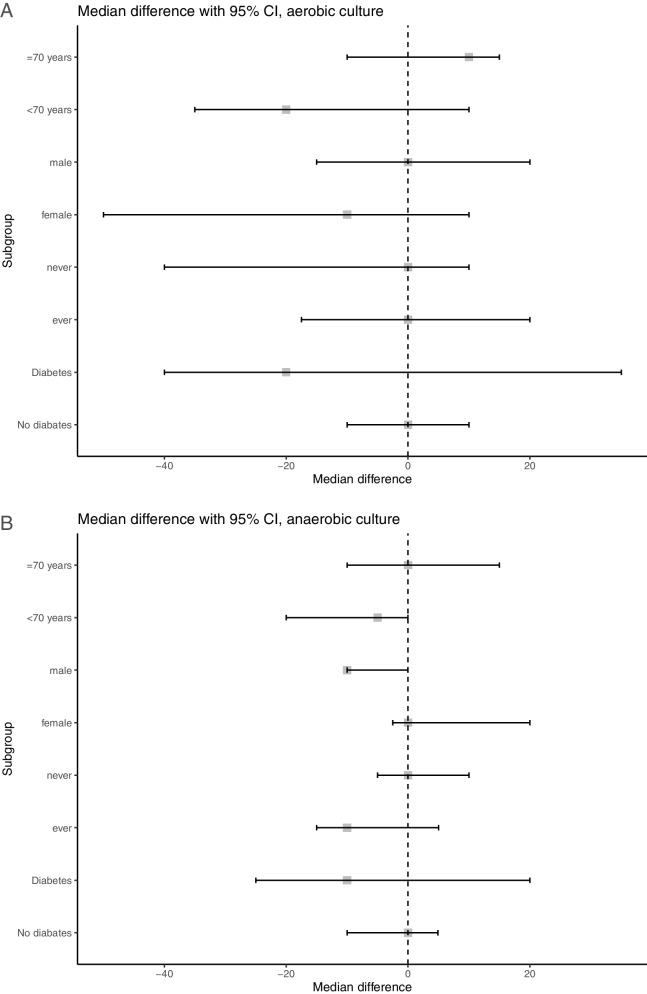
Forest plots of subgroup analyses of colony forming unit counts in aerobic culture (A) and anaerobic culture (B)

The median CFU counts of aspiration needle wash samples in aerobic culture – the primary outcome – were not significantly different between the chlorhexidine mouthrinse group and the usual care group (10 CFU/mL vs 20 CFU/mL, *P* = 0.70; Table 4). There were no significant differences between the groups with regard to secondary outcomes, including median CFU counts in anaerobic culture (*P* = 0.41) and fever within 24 h after EBUS-TBNA (11.8% vs 5.6%; *P* = 0.31). Infectious complications within 4 weeks after EBUS-TBNA did not occur during the course of this study. We performed a sensitivity analysis of 95 patients who underwent EBUS-TBNA with one aspiration needle, whereby the median CFU counts in aerobic and anaerobic culture were not different between the two groups (e-Table 2). We also conducted subgroup analyses for CFU counts in aerobic and anaerobic culture, which did not show any specific subgroup in favor of chlorhexidine mouthrinse (Fig. 2). All patients in the intervention group were adherent with chlorhexidine mouthrinse. Adverse events related to chlorhexidine mouthrinse were not observed.

**Table 4:** Primary and Secondary Outcomes

	Chlorhexidine Mouthrinse (*n* = 51)	Usual Care (*n* = 55)	*P* Value^a^	Median difference (95% CI)^b^
Primary outcome				
CFU counts in aerobic culture, CFU/mL	10 (10–40)	20 (10–40)	0.70	−10 (−20 to 10)
Secondary outcomes				
CFU counts in anaerobic culture, CFU/mL	0 (0–13)	0 (0–20)	0.41	0 (−10 to 0)
Fever within 24 hours after EBUS-TBNA (*n* = 105)^c^	6 (11.8)	3 (5.6)	0.31	
Infectious complications within 4 weeks after EBUS-TBNA (*n* = 105)^d^	0 (0)	0 (0)		

Data are presented as number (%) or median (interquartile range). *CFU* Colony forming unit, *EBUS-TBNA* Endobronchial ultrasound-guided transbronchial needle aspiration

^a^*P*-value from the Mann–Whitney *U* test or Fisher’s exact test. ^b^CI from a bootstrap approach using the percentile method. ^c^One participant was excluded from the analysis owing to loss of 24-hour follow-up. ^d^One participant was excluded from the analysis owing to loss of 4-week follow-up

### Bacterial identification of needle wash samples

**Fig. 3:**
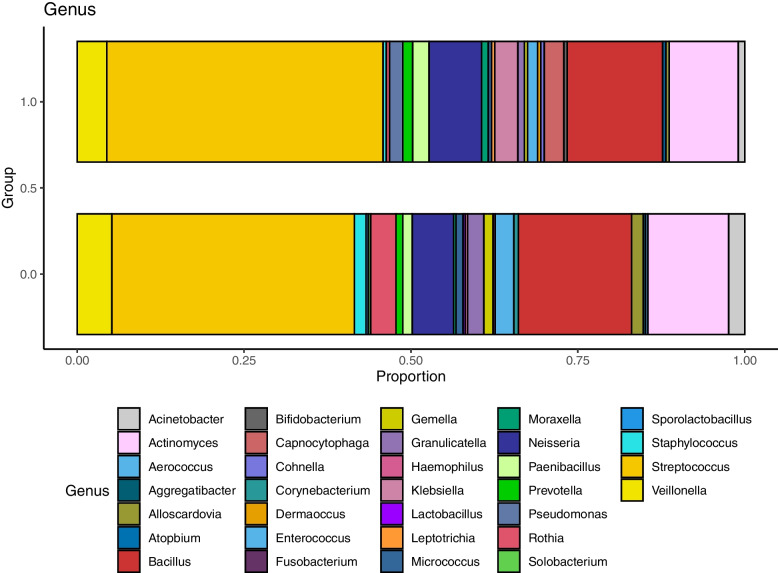
Relative abundance of bacterial genera from needle wash samples. A total of 203 bacteria in the chlorhexidine mouthrinse group and 289 bacteria in the usual care group were identified by matrix-assisted laser desorption ionization time-of-flight mass spectrometry. The dotted boxes represent the genera of oropharyngeal commensal bacteria

Oropharyngeal commensal bacteria were identified in 20 (39.2%) of 51 patients in the chlorhexidine mouthrinse group and in 27 (49.1%) of 55 patients in the usual care group, which was not significantly different (*P* = 0.31; Table 5). Bacteria other than oropharyngeal commensals were found in 23 (45.1%) patients in the chlorhexidine mouthrinse group and 31 (56.4%) in the usual care group (*P* = 0.25). Table 5 and e-Table 3 show details of bacterial genera and species cultured from the needle wash samples on a per-patient basis. A total of 203 bacteria in the chlorhexidine mouthrinse group and 289 bacteria in the usual care group were identified at the species or genus level. Fig. 3 shows the relative abundance of identified bacteria at the genus level, among which the genus *Streptococcus* was the most common in both groups.

**Table 5:** Details of Bacterial Genera from Needle Wash Samples (Per-Patient Analysis)

	Chlorhexidine Mouthrinse (*n* = 51)	Usual Care (*n* = 55)	*P* Value
Oropharyngeal commensal bacteria	20 (39.2)	27 (49.1)	0.31
*Streptococcus* spp.	15 (29.4)	21 (38.2)	
*Actinomyces* spp.	10 (19.6)	14 (25.5)	
*Veillonella* spp.	8 (15.7)	10 (18.2)	
*Neisseria* spp.	7 (13.7)	11 (20.0)	
*Rothia* spp.	1 (2.0)	7 (12.7)	
*Granulicatella* spp.	2 (3.9)	4 (7.3)	
*Prevotella* spp.	2 (3.9)	3 (5.5)	
*Gemella* spp.	1 (2.0)	4 (7.3)	
*Moraxella* spp.	2 (3.9)	1 (1.8)	
*Alloscardovia* spp.	1 (2.0)	2 (3.6)	
*Micrococcus* spp.	1 (2.0)	2 (3.6)	
*Capnocytophaga* spp.	2 (3.9)	0 (0.0)	
*Corynebacterium* spp.	0 (0.0)	2 (3.6)	
*Atopobium* spp.	1 (2.0)	0 (0.0)	
*Bifidobacterium* spp.	1 (2.0)	0 (0.0)	
*Klebsiella* spp.	1 (2.0)	0 (0.0)	
*Leptotrichia* spp.	1 (2.0)	0 (0.0)	
*Aggregatibacter* spp.	0 (0.0)	1 (1.8)	
*Fusobacterium* spp.	0 (0.0)	1 (1.8)	
*Haemophilus* spp.	0 (0.0)	1 (1.8)	
*Lactobacillus* spp.	0 (0.0)	1 (1.8)	
*Solobacterium* spp.	0 (0.0)	1 (1.8)	
Other bacteria	23 (45.1)	31 (56.4)	0.25
*Bacillus* spp.	16 (31.4)	25 (45.5)	
*Paenibacillus* spp.	5 (9.8)	4 (7.3)	
*Staphylococcus* spp.	1 (2.0)	5 (9.1)	
*Enterococcus* spp.	2 (3.9)	3 (5.5)	
*Acinetobacter* spp.	1 (2.0)	2 (3.6)	
*Cohnella* spp.	1 (2.0)	0 (0.0)	
*Dermacoccus* spp.	1 (2.0)	0 (0.0)	
*Pseudomonas* spp.	1 (2.0)	0 (0.0)	
*Aerococcus* spp.	0 (0.0)	1 (1.8)	
*Sporolactobacillus* spp.	0 (0.0)	1 (1.8)	

Data are presented as number of patients (%)

## Discussion

In this RCT investigating the effect of chlorhexidine mouthrinse on the prevention of microbial contamination during EBUS-TBNA, chlorhexidine mouthrinse did not reduce CFU counts of aspiration needle wash samples. Results of sensitivity and subgroup analyses were consistent with this finding. The incidence of fever within 24 hours following EBUS-TBNA was not different between the groups. Infectious complications within 4 weeks after the procedure were not observed.

Previous studies have suggested that postprocedural infection is attributed to contamination of aspiration needles with oropharyngeal commensal bacteria during EBUS-TBNA [18, 19]. Contamination of the working channel of the EBUS bronchoscope could occur during its passage through the oropharynx, and the sterile aspiration needle could be contaminated by oropharyngeal commensal bacteria when it passes through the working channel of the EBUS bronchoscope. During transtracheal or transbronchial passage of the contaminated needle, microorganisms could be directly inoculated into punctured LNs. Several studies reported that contamination of aspiration needles with oropharyngeal commensal bacteria is common [8, 15]. In one study, needle wash cultures were positive in 35% of patients undergoing EBUS-TBNA [8]. In another study showing that endobronchial intubation could prevent contamination by oropharyngeal bacteria during EBUS-TBNA, needle wash cultures were positive in all patients without endobronchial tubes but in only 3% of those with the tubes [15]. Thus, we speculated that oral hygiene has a role as a preventive strategy to reduce infectious complications following EBUS-TBNA. Chlorhexidine mouthrinse has been commonly used not only in dental practice but also in critical care to prevent ventilator-associated pneumonia [20]. Moreover, a previous RCT showed that chlorhexidine mouthrinse before gastroscopy was effective in reducing microbial contamination of the endoscope, resulting in an 88% reduction of the median CFU count of wash samples from the working channel of the endoscope [16]. In this context, we investigated the effect of chlorhexidine mouthrinse on the reduction of microbial contamination during EBUS-TBNA. Chlorhexidine mouthrinse before EBUS-TBNA did not result in a statistically significant reduction in CFU counts of aspiration needle wash samples. However, we performed quantitative cultures of aspiration needle wash samples and bacterial identification using MALDI-TOF MS instead of the traditional technique of biochemical identification, adding strength to the methods used in this RCT.

Potential risk factors for infectious complication after EBUS-TBNA include target lesions with necrotic, cystic, or avascular features and the performance of EUS-B-FNA [10, 18, 19, 21]. The proposed mechanism of infective complications is that decreased blood flow through necrotic lesions could compromise bacterial clearance [19], and repeated puncture by aspiration needles via the esophagus could inoculate esophageal commensal bacteria into the mediastinal target lesions [21]. Although other patient and procedural characteristics in this RCT including the performance of EUS-B-FNA were not significantly different between the chlorhexidine mouthrinse group and the usual care group, the patients with target lesions with coagulation necrosis sign on ultrasound or aspirates with pus-like material were more common in the chlorhexidine mouthrinse group (19.6% vs 5.5%; *P* = 0.03; Table 2). The chlorhexidine mouthrinse patients with higher risk of postprocedural infection showed slightly lower CFU counts in aerobic culture, which did not reach statistical significance, suggesting the possibility of its role in reducing microbial contamination during EBUS-TBNA. However, further analyses were limited because such characteristics of target lesions were post-randomization measures.

Although infectious complications were not observed in this study, we found that bacteria identified from needle wash samples were consistent with common causative bacteria of infectious complications after EBUS-TBNA. In this study, oropharyngeal commensal bacteria were found in 39.2% of patients in the chlorhexidine mouthrinse group and in 49.1% of patients in the usual care group, and the genus *Streptococcus* was the most abundant in both groups. Oropharyngeal commensal bacteria such as the genera *Streptococcus*, *Actinomyces*, *Gemella*, and *Prevotella* were frequently reported as pathogens, and genus *Streptococcus* was identified as the pathogen in 14 of 29 cases of mediastinal infectious complications after EBUS-TBNA [14].

Bacteria that are not generally considered oropharyngeal commensal bacteria were identified in half of the patients in both groups. This could be explained in three ways. First, they could be transitory species in the oropharynx. Second, they could shift from transitory species to colonizers in the oropharynx according to oral health and immune status [22]. As immunocompromised individuals were excluded from the study, poor oral hygiene could be the reason for the shift. The risk factors for periodontal disease include male sex, smoking, and diabetes [23], which were frequently observed in the study patients. Third, bacteria at the lower respiratory tract could contaminate the aspiration needle after being deployed from the sheath.

Our study had several limitations. First, as the sample size was estimated on the basis of an RCT in patients undergoing gastroscopy with or without chlorhexidine mouthrinse [16], it was insufficient to determine the effects of chlorhexidine mouthrinse during EBUS-TBNA. There was no provision for interim analysis in the study protocol [11]. Second, the primary outcome, the CFU count of needle wash samples, was a surrogate marker for the risk of infectious complications associated with EBUS-TBNA. However, adequately powered studies with the primary outcome of infective complications itself would be difficult to perform considering the low incidence of infective complications following this procedure. Third, wash samples obtained from the inner channel of the EBUS bronchoscope rather than the aspiration needle could be another surrogate marker in this study. Fourth, although we excluded patients using antiseptic mouthrinse, oral hygiene was not examined in the current study. However, subgroup analyses according to risk factors for periodontal disease such as age, sex, smoking status, and the presence of diabetes did not show differences between the subgroups. Fifth, the findings of this study may not be generalized to mouthrinse with different concentrations of chlorhexidine and different frequencies and periods of rinsing. Chlorhexidine shows different effects at different concentrations – this antimicrobial agent is bacteriostatic at low concentrations, whereas it is bactericidal at higher concentrations [24].

## Conclusions

In conclusion, chlorhexidine mouthrinse did not reduce CFU counts of needle wash samples of EBUS-TBNA, nor did it affect the incidence of fever following EBUS-TBNA. Large-scale studies are needed to further validate these findings.

## Supplementary Information

**Additional file 1.**

## Data Availability

The datasets analyzed during the current study are not publicly available to protect the participants’ anonymity. But are available from the corresponding author on reasonable request.

## Abbreviations

CFU: Colony forming unit
EBUS-TBNA: Endobronchial ultrasound-guided transbronchial needle aspiration;
EUS-B-FNA: Transesophageal bronchoscopic ultrasound-guided fine-needle aspiration
LN: Lymph node
MALDI-TOF MS: Matrix-assisted laser desorption ionization time-of-flight mass spectrometry
RCT: Randomized controlled trial
