# Correction: Effect of chlorhexidine Mouthrinse on prevention of microbial contamination during EBUS-TBNA: a randomized controlled trial

**DOI:** 10.1186/s12885-023-10505-1

**Published:** 2023-01-23

**Authors:** Na Young Kim, Jae Hyeon Park, Jimyung Park, Nakwon Kwak, Sun Mi Choi, Young Sik Park, Chang-Hoon Lee, Jaeyoung Cho

**Affiliations:** 1 Division of Pulmonary and Critical Care Medicine, Department of Internal Medicine, Seoul National University Hospital, Seoul, Republic of Korea; 2 Department of Laboratory Medicine, Seoul National University Hospital, Seoul, Republic of Korea; 3 Department of Laboratory Medicine, Seoul National University College of Medicine, Seoul, Republic of Korea; 4 Department of Internal Medicine, Seoul National University College of Medicine, 101 Daehak-ro, Jongno-gu, Seoul, 03080 Republic of Korea

**Correction: BMC Cancer 22, 1334 (2022)**

**https://doi.org/10.1186/s12885-022-10442-5**

Following publication of the original article [1], the authors reported errors in Figs. 2 and 3. The incorrect version of the figures were used. figuresThis correction article includes the updated versions of both figures.

**Fig. 2:**
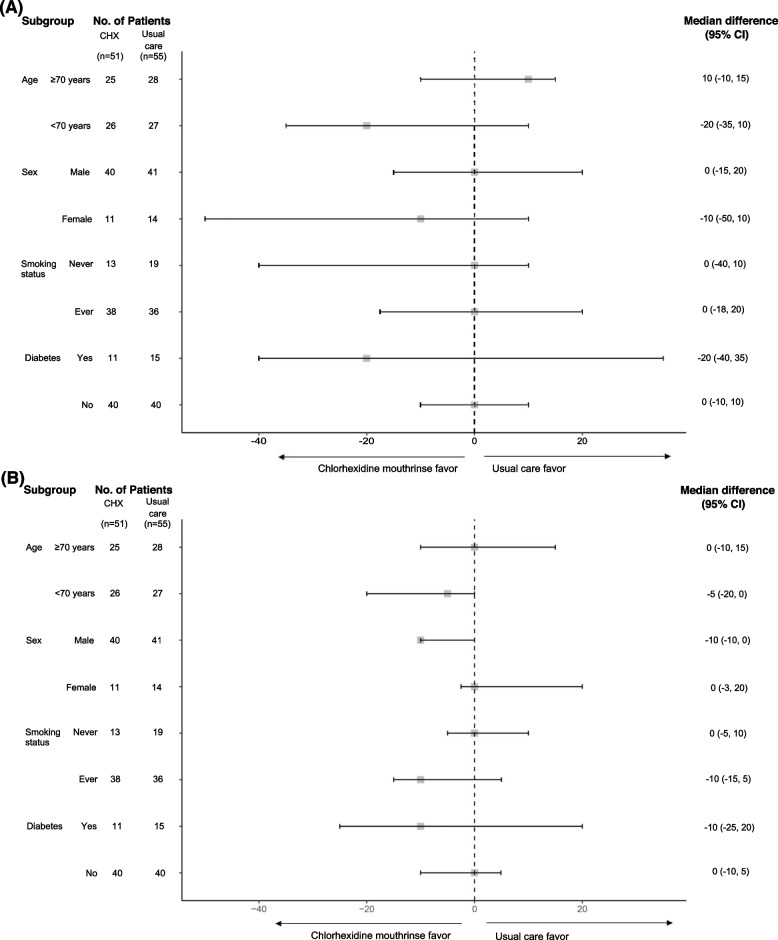
Forest plots of subgroup analyses of colony forming unit counts in aerobic culture (**A**) and anaerobic culture (**B**)

**Fig. 3:**
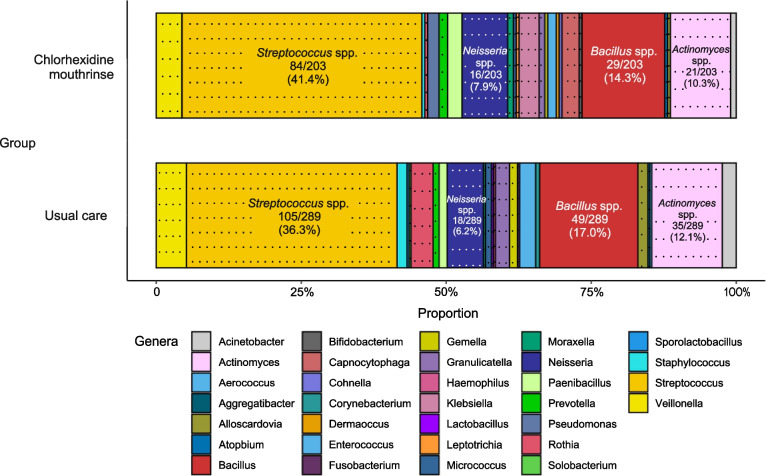
Relative abundance of bacterial genera from needle wash samples. A total of 203 bacteria in the chlorhexidine mouthrinse group and 289 bacteria in the usual care group were identified by matrix-assisted laser desorption ionization time-of-flight mass spectrometry. The dotted boxes represent the genera of oropharyngeal commensal bacteria

Further to this, the authors would like to correct the following:

1) “mouthrinse” in the article title should be in lowercase

2) **Methods**, *Procedures*, second paragraph: The sentence should read: "Following mediastinal evaluation using EBUS, TBNA was performed at the designated lymph nodes (LNs) or masses with a dedicated 22-gauge aspiration needle.", instead of "Following mediastinal evaluation using EBUS-TBNA was performed at the designated lymph nodes (LNs) or masses with a dedicated 22-gauge aspiration needle."

"EBUS-TBNA → EBUS, TBNA"

3) **Results**, *Characteristics of participants and procedure*, third paragraph: The sentence “Cytopathology examinations revealed that 24.5 and 17.9% of the LNs or masses were malignant in the chlorhexidine group and usual care group, respectively.” should be corrected to: “Cytopathology examinations revealed that 24.5% and 17.9% of the LNs or masses were malignant in the chlorhexidine group and usual care group, respectively.”

The '%' sign was omitted after the number 24.5.

"24.5 → 24.5%"

4) In the **abbreviations** section, the semicolon was duplicated before the entry “EUS-B-FNA”.

The original article [1] has been corrected.
