# Supplementary material for: Effect of chlorhexidine Mouthrinse on prevention of microbial contamination during EBUS-TBNA: a randomized controlled trial
Source: BMC Cancer. 2022 Dec 20;22:1334. doi: 10.1186/s12885-022-10442-5 (PMC9764697; doi:10.1186/s12885-022-10442-5)
Supplement: Supplementary file 1 — Additional file 1. [file 12885_2022_10442_MOESM1_ESM.docx]

**Supplemental material**

**Effect of Chlorhexidine Mouthrinse on Prevention of Microbial Contamination During EBUS-TBNA: A Randomized Controlled Trial**

Na Young Kim, MD, Jae Hyeon Park, MD, Jimyung Park, MD, Nakwon Kwak, MD, Sun Mi Choi, MD, Young Sik Park, MD, Chang-Hoon Lee, MD, PhD, Jaeyoung Cho, MD, PhD

e-Table 1. Diagnostic Performance of EBUS-TBNA in Detecting Nodal Metastases

|  | Final surgical diagnosis | | |
| --- | --- | --- | --- |
| EBUS-TBNA Results | Malignant | Benign | Total |
| Malignant | 5 | 0 | 5 |
| Benign | 7 | 66 | 73 |
| Total | 12 | 66 | 78^a^ |

Data are presented as number.

^a^Among 44 of 106 patients who underwent lung resection with mediastinal lymph node dissection, a total of 78 lymph nodes were sampled. The sensitivity, specificity, positive predictive value, negative predictive value, and diagnostic accuracy of endobronchial ultrasound-guided transbronchial needle aspiration (EBUS-TBNA) in detecting nodal metastasis was 42% (95% CI, 15–72%), 100% (95–100%), 100% (48–100%), 90% (81–96%), and 91% (82–96%), respectively.

e-Table 2. Sensitivity Analysis in Which Only Participants Who Underwent EBUS-TBNA With One Aspiration Needle Were Included (n = 95)

|  | Chlorhexidine Mouthrinse  (n = 44) | Usual Care  (n = 51) | *P* Value^a^ | Median difference (95% CI)^b^ |
| --- | --- | --- | --- | --- |
| Primary outcome |  |  |  |  |
| CFU counts in aerobic culture, CFU/mL | 10 (10–35) | 10 (10–40) | 0.71 | 0 (–20 to 10) |
| Secondary outcome |  |  |  |  |
| CFU counts in anaerobic culture, CFU/mL | 0 (0–10) | 0 (0–20) | 0.44 | 0 (–10 to 5) |

Data are presented as median (interquartile range).

^a^*P*-value from the Mann–Whitney *U* test.

^b^CI from a bootstrap approach using the percentile method.

CFU = colony forming unit; EBUS-TBNA = endobronchial ultrasound-guided transbronchial needle aspiration.

e-Table 3. Details of Bacterial Species from Needle Wash Samples (Per-Patient Analysis)

|  | Chlorhexidine  Mouthrinse  (n = 51) | Usual Care  (n = 55) |
| --- | --- | --- |
| Oropharyngeal commensal bacteria | | |
| *Streptococcus* spp. |  |  |
| *S. parasanguinis* | 6 | 16 |
| *S. salivarius* | 6 | 6 |
| *S. mitis* | 5 | 3 |
| *S. oralis* | 5 | 4 |
| *S. vestibularis* | 4 | 2 |
| *S. anginosus* | 1 | 4 |
| *S. pneumoniae* | 1 | 1 |
| *S. peroris* | 0 | 3 |
| *S. constellatus* | 0 | 2 |
| *S. pseudopneumoniae* | 0 | 1 |
| *S. infantis* | 0 | 1 |
| *S. sanguinis* | 0 | 1 |
| *S. gordonii* | 1 | 0 |
| *Streptococcus* spp.^a^ | 9 | 14 |
| *Actinomyces* spp. |  |  |
| *A. odontolyticus* | 5 | 9 |
| *A. graevenitzii* | 1 | 2 |
| *A. oris* | 2 | 1 |
| *Actinomyces* spp.^a^ | 5 | 12 |
| *Veillonella* spp. |  |  |
| *V. atypica* | 6 | 8 |
| *V. parvula* | 0 | 1 |
| *V. dispar* | 0 | 1 |
| *Veillonella* spp.^a^ | 2 | 3 |
| *Neisseria* spp. |  |  |
| *N. subflava* | 4 | 4 |
| *N. flavescens* | 1 | 4 |
| *N. perflava* | 0 | 1 |
| *Neisseria* spp.^a^ | 4 | 4 |
| *Rothia* spp. |  |  |
| *R. mucilaginosa* | 0 | 2 |
| *R. aeria* | 0 | 1 |
| *Rothia* spp.^a^ | 1 | 5 |
| *Granulicatella* spp. |  |  |
| *G. adiacens* | 1 | 2 |
| *Granulicatella* spp.^a^ | 1 | 3 |
| *Prevotella* spp. |  |  |
| *P. melaninogenica* | 0 | 1 |
| *P. salivae* | 0 | 1 |
| *Prevotella* spp.^a^ | 2 | 1 |
| *Gemella* spp. |  |  |
| *G. haemolysans* | 0 | 3 |
| *G. sanguinis* | 1 | 0 |
| *Gemella* sp.^a^ | 0 | 1 |
| *Moraxella* spp. |  |  |
| *M. osloensis* | 0 | 1 |
| *Moraxella* spp.^a^ | 2 | 0 |
| *Alloscardovia* spp. |  |  |
| *A. omnicolens* | 1 | 2 |
| *Alloscardovia* sp.^a^ | 0 | 1 |
| *Micrococcus* spp. |  |  |
| *M. luteus* | 1 | 2 |
| *Capnocytophaga* spp. |  |  |
| *C. gingivalis* | 2 | 0 |
| *Capnocytophaga* sp.^a^ | 1 | 0 |
| *Corynebacterium* spp. |  |  |
| *C. durum* | 0 | 1 |
| *C. striatum* | 0 | 1 |
| *Atopobium* sp. | 1 | 0 |
| *Bifidobacterium* spp. |  |  |
| *B.* *infantis* | 1 | 0 |
| *Klebsiella* spp. |  |  |
| *K. pneumoniae* | 1 | 0 |
| *Leptotrichia* sp. | 1 | 0 |
| *Aggregatibacter* sp. | 0 | 1 |
| *Fusobacterium* sp. | 0 | 1 |
| *Haemophilus* spp. |  |  |
| *H.* *parainfluenzae* | 0 | 1 |
| *Lactobacillus* sp. | 0 | 1 |
| *Solobacterium* sp. | 0 | 1 |
| Other bacteria |  |  |
| *Bacillus* spp. |  |  |
| *B. infantis* | 6 | 9 |
| *B. halosaccharovorans* | 2 | 2 |
| *B. simplex* | 1 | 2 |
| *B. flexus* | 1 | 1 |
| *B. megaterium* | 2 | 0 |
| *B. circulans* | 0 | 1 |
| *B. horikoshii* | 0 | 1 |
| *B. idriensis* | 0 | 1 |
| *B. licheniformis* | 0 | 1 |
| *B. mojavensis* | 0 | 1 |
| *Bacillus* spp.^a^ | 11 | 18 |
| *Paenibacillus* spp. |  |  |
| *P. humicus* | 0 | 1 |
| *P. amylolyticus* | 1 | 0 |
| *P. timonensis* | 1 | 0 |
| *Paenibacillus* spp.^a^ | 3 | 3 |
| *Staphylococcus* spp. | 1 | 5 |
| *Enterococcus* spp. |  |  |
| *E. faecalis* | 1 | 1 |
| *E. faecium* | 0 | 1 |
| *Enterococcus* spp.^a^ | 2 | 2 |
| *Acinetobacter* spp. |  |  |
| *A. baumannii* | 1 | 2 |
| *Acinetobacter* spp.^a^ | 0 | 2 |
| *Cohnella* sp. | 1 | 0 |
| *Dermacoccus* sp. | 1 | 0 |
| *Pseudomonas* spp. |  |  |
| *P. aeruginosa* | 1 | 0 |
| *Aerococcus* spp. |  |  |
| *A. viridans* | 0 | 1 |
| *Sporolactobacillus* sp. | 0 | 1 |

Data are presented as number of patients.

^a^The bacteria were identified by matrix-assisted laser desorption ionization time-of-flight mass spectrometry (MALDI-TOF MS) using MALDI Biotyper (Bruker Daltonics, Bremen, Germany) with 6903 main spectra library. MALDI-TOF MS identifications were classified using score values proposed by the manufacturer: a score value of ≥2 indicated species identification, a score value between 1.7 and 1.999 indicated genus identification, and a score value of <1.7 indicated unreliable identification. These indicated bacteria were identified at the genus level by MALDI-TOF MS.
